# Medical and Physical Intelligence

**Published:** 1801-08

**Authors:** 


### MEDICAL AND PHYSICAL INTELLIGENCE.

### [ FOREIGN AND DOMESTIC. ]

*Establishment for the Preparation of Artificial Mineral Waters at Paris.*—There exifts at prefent in Paris, an eftablifhment for the preparation of mineral waters, which is certainly unique in its kind. It has been inftituted by *Citizen Paul* and *Company* under whofe direction this manufactory of mineral waters is carried on with great fuccefs and profit. The Society of Medicine at Paris appointed a committee, confifting of the *Citizens Chaussier, Delunel, Tourcy, Jossee, Pelletier*, &c. in order to give an accurate account of that eftablifhment to the fociety, and to examine the nature of an undertaking, which may prove very ufeful to the community, as by that means all the beneficial remedies which nature has placed at great diftances are united in one place, and procured in a lefs expenfive way than is otherwife the cafe. According to the report given by thofe gentlemen, the preparation of thefe mineral waters is executed upon a large fcale with the greateft exactnefs, and the machines invented by *Citizen Paul*, for the purpofe of combining the gaffes with the water, have the double perfection of being very fimple and perfectly calculated for the purpofe. Particular attention has been employed for purifying the water that is to be mineralized by filtration, and to difengage it from all heterogenous particles. It paffes five cylinders of lead filled with fand, and placed in graduated heights, by which the water being reforbed, comes out again in the higheft degree of limpidnefs, which makes thefe waters more palatable than the natural ones. The chemical proceedings are extremely accurate. The gaffes which admit it, are purified by making them pafs by degrees four veffels full of water before they come into the laft, which is hermetically fhut, where the combination proceeds. The waters containing carbonic acid in their compofition, are generally impregnated with a larger proportion of it than the waters that are produced by nature; but it muft be confidered, that a great deal of it evaporates when the bottle is opened, and the water poured into the glafs to drink, and part of it is alfo difcharged from the ftomach. *Citizen Paul*, however, is able to charge his waters with any quantity of gas that may be thought proper, according to the prefcription of the phyfician. In order to make his mineral waters imitate the natural ones as nearly as poffible, *Citizen Paul* obferves, moft fcrupuloufly, the analyfes of *Bergman* and others, who have adopted the accurate method of that great chemift. Thofe analyfes which have not been thought to be made accurately enough, were repeated by that great operator, *Citizen Vauquelin*, for the ſake of rendering the compoſition of theſe artificial mineral waters as natural as poſſible. The following table will inform the reader of the proportion of the conſtituents in the different waters that are prepared. Each bottle of 20 ounces (6,11 hectogrammes), contains the following doſes:

1. *Strong water of Seltz.*

Carbonic acid diſengaged by effervefcence, 5 *times* its volume

Magneſia 2 grains.

Carbonat of ſoda 4 gr.

Muriat of ſoda 22 gr.

2. *Sweet water of Seltz.*

Carbonic acid diſengaged by fire, 4 *times* its volume.

The three ſalts in the ſame proportion as in the former.

3. *Water of Spa.*

Carbonic acid, by effervefcence, 5 *times* its volume.

Magneſia 4 gr.

Carbonat of ſoda 2 gr.

Muriat of ſoda $\frac{1}{3}$ gr.

Carbonat of iron $\frac{1}{2}$ gr.

The *ſtrong water at Spa* has only the double proportion of the Carbonat of iron.

4. *Water of Sedlitz.*

Carbonic acid, 5 *times* its volume.
Sulphat of magneſia, 144 gr.

5. *Water of Vichy.*

Carbonic acid by effervefcence, *twice* its volume.

Carbonat of lime 2 gr.

Carbonat of magneſia $\frac{1}{2}$ gr.

Carbonat of iron $\frac{1}{10}$ gr.

Carbonat of ſoda 24 gr.

Sulphat of ſoda 4 gr.

Muriat of ſoda 4 gr.

6. *Water of Buſſang.*

Carbonic acid, 3 *times* its volume.

Carbonat of ſoda 6 gr.

Carbonat of iron $\frac{1}{2}$ gr.

7. *Water of Vals.*

Carbonic acid, 3 *times* its volume.

Muriat of ſoda 15 gr.

Sulphat of iron $\frac{1}{2}$ gr.

Sulphat of argil $\frac{1}{2}$ gr.

Carbonat of iron $\frac{3}{4}$ gr.

8. *Water of Contrexeville*

Carbonic acid, *twice* its volume.
Sulphat of lime 6 gr.
Carbonat of lime 4 gr.

9. *Water of Balaruc.*

Carbonic acid, *twice* its volume.

Lime 4 gr.

Muriat of ſoda 12 gr.

Carbonat of potaſh 4 gr.

10. *Water of Plombieres.*

Carbonic acid, $\frac{1}{20}$ of its volume.

Sulphat of lime 3 gr.

Carbonat of lime 2 gr.

Sulphat of magneſia 1 gr.

11. *Alkaline Gaſous Water.*

Carbonic acid, 6 *times* its volume.

Carbonat of potaſh 144 gr.

12. *Hydrogenated water.*

Hydrogen gas, $\frac{1}{2}$ its volume.

13. *Hydro-carbonated water.*

Hydro-carbonated gas, $\frac{2}{3}$ its volume.

14. *Weak Hydro-sulphurated water.*

Hydrogen gas, *half* its volume.

Hydrogen ſulphurated gas, $\frac{1}{32}$ its volume.

15. *Strong*

15. *Strong Hydro-sulphurated water*
Hydrogen gas, *half* its volume.
Hydrogen fulphurated gas ¼ its volume.

16. *Oxygenated water.*
Oxygen gas, *half* its volume.

Such is the compofition of the different artificial mineral waters which are made here, and it has been found perfectly agreeing with the analyfis that thofe gentlemen have undertaken. The ufe of thofe waters' which are imitated from nature is fufficiently known, but it may not be improper to add fomething of the medical properties of thofe that are only produced by art, viz. of *the alkaline gaseous, hydrogenated waters,* &c. The *alkaline gaseous water* has been much recommended in the calculus and gravel of the bladder; and though it is not able to diffolve the calculus, as has been by fome afferted, yet it greatly diminifhes the pains that attend thofe complaints. Three or four glaffes of it, with a little milk, ought to be drank every morning within the fpace of fix hours. It is likewife of ufe in other affections of the bladder. *The hydrogenated water* acts as an antifpafmodic and soporific. *The hydrosulphurated water* has a great fimilarity with the fulphurated waters of hot wells by its hepatic fmell and tafte. It is diaphoretic and refolvent, and may be employed in obftructions of the vifcera, tumours, &c.

Profeffor *Odier*, at Geneva, relates feveral cafes that were cured by this water, and among others, of a woman, who had for two years a painful tumour or fcirrhus in the breaft, which the furgeons intended to extirpate, and at the fame time an enormous wen *(goitre)*, for the laft of which fhe began to take the hydro-fulpurated water; and having continued it for above two months, fhe was cured at the fame time of her wen and the tumour in the breaft.—The ftrong hydro-fulphurated waters employed in baths and lotions are extremely ferviceable in all pforic difeafes and inveterate ulcers. The art of preparing the *oxygenated water* is entirely owing to *Citizen Paul*, a difcovery which is very important, and may prove ufeful both to arts and medicine. The oxygen gas is not very intimately combined with the water, but eafily difengaged from it; however, a fufficient quantity is retained in it to produce fenfible effects upon the animal œconomy, particularly if proper care be taken to prevent its evaporation. It increafes and forces the appetite, and it has been found of great benefit in fpafms of the ftomach, humid afthma, dropfy, periodic and nervous affections, which even refifted the bark and the moft efficacious antifpafmodics, in lingering convalefcences, and, in fhort, in all cafes where it is required to increafe the tone of the organs and to ftimulate the circulation. It is given by glaffes every two hours. Sometimes it produces dyfuries, on which account we fhould begin with fmall quantities.

The fabrication of all thefe waters is eftablifhed in a very large houfe, in which *Citizen Paul* has at the fame time endeavoured to unite every convenience for thofe who defire to ufe the waters in baths.

baths. In one wing of the building are feveral bathing rooms for men, and in another oppofite to it, thofe for women, moft of which are provided with a bed. Every fort of bath may be had here, fimple or mineral, warm or cold, &c. In the upper ftory are feveral commodious apartments, to lodge any patient who might wifh, on account of health, to live near the baths. They are furnifhed with every neceffary, and their refidence rendered as comfortable as poffible. A meadow near the houfe is intended to feed cows, goats, and affes, for the ufe of thofe patients to whom milk might be neceffary. There belongs befides to the houfe, a garden communicating with the delightful garden of Tivoli, which affords to thofe who ufe the waters, a moft pleafant walk.

Every kind of gas is likewife made by the fame company.

---

VAUQUELIN *on the Gadolinit.* It is about feven years ago fince Mr. GADOLIN difcovered a new earth, in a foffil found near *Ytterby* in *Sweden*, which he therefore called *Yttria*. It was afterwards fubmitted to a new analyfis by Mr. EKEBERG; according to whofe experiments, this new earth is contained in the foffil, in proportion of 0,47 to 100. The foffil itfelf is of a black colour, and its powder a blackifh grey; at the place where it is broke, it appears vitreous; its fpecific weight is, after *Cit.* HAÜY, 4,049. *Cit.* VAUQUELIN has alfo analyfed this foffil, which he calls GADOLINITS, with acids and potafh, and the proportion of its conftituents are the following;

| | |
|---|---|
| 1. Siliceous earth - - | 25. 5 |
| 2. Oxyd of iron - - | 25 |
| 3. Oxyd of manganefe - | 2 |
| 4. Lime - - - - - - | 2 |
| 5. New earth or Yttria - | 35 |
| | 89. 5 |
| Lofs, | 10. 5 |
| | 100 |

The following are the properties of this new earth. 1. It is quite white, though it is with difficulty obtained in this ftate, on account of the oxyd of manganefe that adheres fo clofely to it. 2. It has neither tafte nor fmell. 3. It is not fufible by itfelf; borax, however, diffolves it, forming with it a white tranfparent glafs, if too great a quantity be not added. 4. It is not perceptibly foluble in fixed cauftic alkalis, on which account it differs from argil and the glucine. 5. It is foluble in the carbonat of ammonia, but about 5 or 6 times greater quantity of it is required to diffolve the Yttria than the glucine. 6. It rapidly combines itfelf with fulphuric acid; and while this combination proceeds, the falt that is hereby formed, cryftallizes in fmall granula, which are but little foluble in water. It has at firft an aftringent and afterwards a fweet tafte, and though it agrees in refpect to this property with the glucine, glucine, it fufficiently differs in other characters. 7. Its combination with nitric acid cryftallizes with difficulty, and its affinity to water fo great, that it is hardly poffible to dry it. Sulphuric acid forms, when poured to a folution of nitrat of Yttria, a precipitate, which is fulphat of Yttria. 8. Its combination with muriatic acid has nearly the fame properties as the nitrat of Yttria. 9. Ammonia precipitates the Yttria from the above three combinations, and likewife the lime and barytes. 10. Oxalic acid, and confequently the oxalate of ammonia, produce precipitates in appearance extremely like that of the muriatic of filver, whereas the glucine forms, with oxalic acid, a very foluble falt. 11. The pruffiat of potafh cryftallized and again diffolved in water, occafions in the folutions of this earth by acids, a white granulated fediment, which it does not produce in the folutions of glucine. 12. Phofphoric acid does not precipitate it from other acids, but the phofphate of foda feparates it in form of white gelatinous floccula. 13. It feems to have a greater affinity with fome acids than the glucine. 14. An infufion of galls precipitates it from its folutions in form of brown floccula.

---

Dr. Handel, of Menz, recommends the following remedy, as a very powerful fedative in tooth-ach, occafioned by corrupted or hollow teeth; upon the application of which, the excruciating pains almoft inftantly ceafe. ℞. *Olei hyosciam, dr. j. Opii thebaici drach. dimid. extract. belladon. camphoræ ana gran. vj. Olei cajeput, tincturæ cantharidum ana gutt. viij. Redigantur in formam opiat.*

---

*On the Analysis of the Honey-stone by Cit. Vauquelin.*—Our readers have been informed, through the medium of this Journal, of the different analyfes that have been made of the honey-ftone, (Germ. *Honigstein; Mellilithas)* by Abich, Lampadius, and Klaproth, the laft of whom has difcovered, that it contained an acid of its own, named by him *Mellilithic acid.* This interefting mineral has been fince fubmitted to a new analyfis by that great chemift, *Citizen Vauquelin,* whofe particular fkill in the chemical analyfis is fufficiently known.

*Two grammes* * of this ftone being reduced to powder, were mixed with four parts of faturated carbonat of potafh, diffolved in a fufficient quantity of water, by which an effervefcence was occafioned without the application of any external heat; but, in order to render the compofition more perfect, the mixture was flightly heated in a fand bath. The liquor being filtrated, when cold, was of a brownifh colour, and left on the paper a brown fubftance, which, after being dryed by the fun, weighed about 0,8 *grammes.* This calcined in a crucible, became white, weighing only about 0,33 *grammes.*

---

* A gramme is about 20 gr. or a fcruple, (gros in French.)

*grammes*. Being mixed with diluted ſulphuric acid, a ſlight efferveſcence was produced, and the mixture was afterwards evaporated to dryneſs. It was then put into water, in which however, only a ſmall quantity diſſolved, the reſt remaining untouched as a white powder. The liquor was evaporated ſo far, that only 3 or 4 grammes remained, to which one drop of ſulphat of potaſh being added, 0,1 gramme of argil, with a little ſulphat of lime, was obtained after the evaporation. *Citizen Vauquelin* now proceeded to enquire into the nature of that white ſubſtance which did not diſſolve in water. For this purpoſe it was boiled with a ſolution of carbonat of potaſh, filtered, waſhed, and examined in the following way:—1. Muriatic acid, diluted with two parts of water, diſſolved it with a ſlight efferveſcence, but the ſolution remained milky. 2. This liquor being filtered, gave with ammonia, a precipitate which much reſembled that which argil affords in the ſame way; but though it did not quite diſſolve, yet the part ſoluble in potaſh was the greateſt, having all the characteriſtics of aluminous earth, or argil. The liquor from which the ammonia had precipitated the argil, gave ſlight precipitates with the carbonat of potaſh and the oxalate of ammonia, which proved its containing ſome lime. The inſoluble part of that ſubſtance weighed about 0,1 *grammes*, and ſeemed to be ſiliceous earth. Having in this manner become acquainted with the nature of the firſt reſiduum, the liquor was examined, which ought to contain the acid of the honey-ſtone united with the potaſh. In hopes that it might yield its baſis to mineral acids, a few drops of nitric acid were added to the liquor, which producing a very ſlight efferveſcence, diſengaged a ſmall quantity of a brown flaky ſubſtance. Some hours after, the acid of the honeyſtone cryſtallized in form of ſmall ſhort priſms with brilliant facets. On finding that in this way the acid may be ſeparated from the potaſh, the liquor was heated, and more nitric acid mixed with it, till it was perceived by its taſte to predominate. Being now filtered, about 1.34 *grammes* of the new acid were obtained by the two cryſtallizations; the properties it ſhowed, were the following: 1. It is of a conſiderable hardneſs, has a ſlight acid taſte, attended with a little bitterneſs, which may be owing to the adherent bituminous particles.—2. A portion of this acid expoſed to the flame of the blow-pipe, puffed up after a previous ſlight detonation, leaving a ſubſtance that eaſily penetrated the coal.—3. Heated in a crucible of platinæ, it puffs up at firſt, but is afterwards reduced to a coal, without producing any oily fumes, and this coal is light, and in a great meaſure alkaline. This acid remains therefore united with a certain quantity of potaſh, notwithſtanding the ſuperabundance of nitric acid that was added to its ſolution, a circumſtance that alſo takes place in the tartarous and oxalic acid, which come by that means into the ſtate of *acidulated* ſalts. 4. It is but little ſoluble. 5. Some *grammes* of it diſſolved in water, were mixed with a ſolution of lime, by which inſtantly a white flaky precipitate was produced, that fell to the bottom; with a ſolution of ſulphat of lime, a ſlight granulated cryſtallized precipitate was occaſioned, which was increaſed, and became became flaky by the addition of a drop of ammonia; with the ſolution of muriate of barytes, a number of needle-like cryſtals were precipitated; with a ſolution of ſilver a white ſhining precipitate was cauſed, that fell down in the ſhape of a fine powder; with a ſolution of lead in nitric acid, a heavy white powder was precipitated; with a ſolution of mercury, it gave a white precipitate, that was blackened by a drop of ammonia added to it.—The reſult of theſe experiments is, that the acid of the honey-ſtone has many properties analogous to thoſe of the acid of ſorrel, and ſeems to differ only by the following diagnoſtics. 1. The precipitate which it occaſions in the ſolution of ſulphat of lime appears not ſo ſoon, and is cryſtalline inſtead of being powdery, like that which is formed by the acidulated oxalat of potaſh. 2. It appears to be leſs acid to the taſte than the acidulated oxalat of potaſh, which, however, depends probably on the nitric acid being not ſufficiently added to its combination with potaſh, in order to deprive it of a ſufficient quantity of that alkali. 3. It puffs up a little ſooner than the acidulated oxalat of potaſh.—The octaëdrous form of the honey-ſtone, ſeems likewiſe to have a ſort of analogy with the oxalic acid. *Citizen Vauquelin*, however, had too ſmall a quantity of the above acid to diſpoſe of, to be able to ſhow by farther experiments the identity of it with the oxalic acid, which he is much inclined to adopt; but if this be confirmed, we ſhould have the oxalic acid in all three *regna naturæ*, viz. as acidulated oxalate of potaſh in ſeveral kinds of vegetables, in that of oxalate of lime in the calculus veſicæ urinariæ, and at laſt in the ſtate of oxalat of argil in the honey-ſtone.

---

Dr. Bobba, of Italy, has preſented to the Medical Society at Paris, ſome ingenious remarks on the cauſe of rickets. It is known that the bones owe their ſolidity to the phoſphat of lime, and that conſequently the cauſe of rickets has been aſcribed to a want of that ſubſtance. However plauſible this theory may be, there are caſes recorded by Morgagni, Portal and Pinel, where a mollification of the bones was obſerved to be complicated with the gout. Such a complication ſeems at firſt ſight to be impoſſible, as one diſeaſe originates in a want, and the other in a ſuperabundance of the phoſphat of lime. This contradiction, however, is but apparent; for, when the bones begin to mollify, we are not always entitled to conclude, that the phoſphat of lime is entirely wanting in the ſyſtem, but it is ſometimes probable, that on account of an inactivity in the veſſels which carry this ſubſtance to the bones, it is directed to other parts, producing arthritic concretions, preternatural oſſifications, &c. Frequently it is depoſited in the urinary ſyſtem, partly from being abſolutely ſuperabundant, partly becauſe any morbific cauſe prevents its being carried to the bones; and it is remarkable, that in almoſt all the diſeaſes of the bones the urine depoſits a calcareous ſediment. There are beſides, ſome rare caſes, where this calcareous matter has deviated to the genitals and urethra, and gives riſe moſt probably to that ſpecies of blenorrhagy, called by SWEDIAUR *arthritic.* By a derivation, therefore, of the phofphat of lime from the bones to the joints, fymptoms of gout are produced, at the fame time a mollification of the bones, which complication is named *arthritis rachitica.* Dr. BOBBA terminates his paper with obferving, that a bad quality of the milk with which children are nourifhed, is likely to be a frequent remote caufe of the rickets, and that a tonic treatment of this difeafe would probably anfwer better than the alkaline treatment, which has been recommended by fome practitioners.

---

*Profeffor Abildgaard*, at Copenhagen, relates in a letter to *Citizen Huzard*, the refults of his experiments for the purpofe of afcertaining the quantity of carbon that is contained in the blood; according to which, he has found it to exift in a greater proportion in the arterial than in the veinous blood. 1. 100 parts of the veinous blood of a horfe have afforded, when dryed at a moderate heat, 26 parts of a dry fubftance that could be pulverized.—2. 100 parts of arterial blood of the fame horfe gave 25 parts of a dry fubftance.—3. For alkalizing after Kirwan's method, one ounce of nitre by detonation, 192 grains of veinous blood were required, and only 160 gr. of arterial blood. 4. One ounce of veinous blood gave after being dryed and decompofed in a clofe veffel, 115 ½ gr. of coal.—5. The fame quantity of arterial blood gave only 87 ½ gr. of coal.—6. For decompofing 480 gr. of nitre, 145 gr. of coal of the veinous blood were required, whereas for this purpofe only, 119 gr. of coal of arterial blood were employed. This experiment, however, is not very exact, as a part of the coal being extremely light, flies away like duft. The coal of arterial blood is lighter than that of veinous.—7. The red part of the blood, feparated from the ferum and the fibrous part, as exactly as this is poffible in the common way, was, after having become dry, tried with nitre, and 130 grains of this red part were requifite for alkalizing the nitre.—8. Of the fibrous part, well feparated from the ferum, 202 gr. have been required for alkalizing the nitre by detonation. This part of the blood, however, detonates with greater vivacity than the reft.

---

Dr. GARNETT intends to deliver Lectures on the Theory and Practice of Medicine, and on the Animal Economy; he alfo propofes to deliver Lectures on Chemiftry, and thofe Branches of Natural Philofophy particularly connected with Medicine. Thefe different Courfes will commence in October next, at his Houfe, No. 51, Great Marlborough Street.

